# Exploring the Need for Medical Futures Studies: Insights From a Scoping Review of Health Care Foresight

**DOI:** 10.2196/57148

**Published:** 2024-10-09

**Authors:** Bertalan Meskó, Tamás Kristóf, Pranavsingh Dhunnoo, Nóra Árvai, Gellért Katonai

**Affiliations:** 1 The Medical Futurist Institute Budapest Hungary; 2 Kálmán Laki Doctoral School of Biomedical and Clinical Sciences University of Debrecen Debrecen Hungary; 3 Institute of Entrepreneurship and Innovation Corvinus University of Budapest Budapest Hungary; 4 Department of Computing Atlantic Technological University Letterkenny, Co. Donegal Ireland; 5 Meducation Hungary Kft Budapest Hungary; 6 Department of Family Medicine Semmelweis University Budapest Hungary

**Keywords:** foresight, futures studies, health care future, medical futures, technology foresight

## Abstract

**Background:**

The historical development and contemporary instances of futures studies, an interdisciplinary field that focuses on exploring and formulating alternative futures, exemplify the increasing significance of using futures methods in shaping the health care domain. Despite the wide array of these methodologies, there have been limited endeavors to employ them within the medical community thus far.

**Objective:**

We undertook the first scoping review to date about the application of futures methodologies and published foresight projects in health care.

**Methods:**

Through the use of the PRISMA-ScR (Preferred Reporting Items for Systematic Reviews and Meta-Analyses extension for Scoping Reviews) method, we identified 59 studies that were subsequently categorized into the following 5 distinct themes: national strategies (n=19), strategic health care foresight (n=15), health care policy and workforce dynamics (n=6), pandemic preparedness and response (n=7), and specialized medical domains (n=12).

**Results:**

Our scoping review revealed that the application of futures methods and foresight has been successfully demonstrated in a wide range of fields, including national strategies, policy formulation, global threat preparedness, and technological advancements. The results of our review indicate that a total of 8 futures methods have already been used in medicine and health care, while there are more than 50 futures methods available. It may underscore the notion that the field is unexploited. Furthermore, the absence of structured methodologies and principles for employing foresight and futures techniques in the health care domain warrants the creation of medical futures studies as a separate scientific subfield within the broad domains of health care, medicine, and life sciences. This subfield would focus on the analysis of emerging technological trends, the evaluation of policy implications, and the proactive anticipation and mitigation of potential challenges.

**Conclusions:**

Futures studies can significantly enhance medical science by addressing a crucial deficiency in the promotion of democratic participation, facilitating interdisciplinary dialogue, and shaping alternative futures. To further contribute to the development of a new research community in medical futures studies, it is recommended to establish a specialized scientific journal. Additionally, appointing dedicated futurists in decision-making and national strategy, and incorporating futures methods into the medical curriculum could be beneficial.

## Introduction

Analyzing the future of medicine and health care has lately become a major focus for medical journals and the life science community owing to the exceptional technological progress witnessed in health care, digital health’s cultural transformation of traditional roles of patients and medical professionals, and the growing importance of preparing for critical changes in the health care ecosystem, which was recently demonstrated by the COVID-19 pandemic.

While “futures studies” has been an established academic discipline for decades, medical professionals and life science researchers have rarely used so-called futures methods in their studies and analyses. However, acknowledging the growing role that a practical domain of futures studies (foresight) plays in shaping the health care landscape and tracking its historical development within this sector can shed light on previous trends and approaches while also establishing a foundation for the emergence and indispensability of medical futures studies as an emerging academic subfield. Textbox 1 describes the related basic terms [1].

**Basic terms.**

Futures field: A broader domain that integrates futures studies and foresight to influence decision-making and strategic planning across different sectors.

Futures studies: An academic field focused on exploring and shaping various possible, probable, feasible, and preferred futures through interdisciplinary methods.

Foresight: The strategic practice of exploring and preparing for possible future scenarios to guide present decisions.

Futures methods: Techniques and tools used in futures studies and foresight to systematically explore, create, and test possible and desirable futures to improve decisions.

According to this paper [1], “futures studies” is classified as an academic subject, whereas foresight is considered a practical domain involving more specific strategic planning based on futures knowledge. Both subjects are part of the broader category known as the futures field.

Within this, rooted in social sciences, “futures studies” is an interdisciplinary field with the primary focus to explore and formulate alternative futures [2,3]. Throughout its multiple decades of development, “futures studies” has become a generally accepted academic discipline since the 1960s. Its merit is visible in the international scientific community, having global professional associations, higher education programs, and prestigious scientific journals. Futurists globally adhere to universally accepted theoretical concepts, methodologies, purposes, tenets, ethical principles, and empirical results. Textbox 2 summarizes its short history.

**History of futures studies.**

“Futures studies” is an interdisciplinary field that is grounded in the social sciences. Its main objective is to explore and construct alternative futures. The primary goal of futures studies is to analyze and control the causal relationships between events and outcomes in the future using a systematic approach that incorporates feedback loops to foster social and technological innovations [4]. Kristóf and Nováky [3] have provided a comprehensive summary of the developmental trajectory of futures studies. Historically, the term “foresight” has been used to refer to the readiness to address future issues. The concept of “foresight” emerged in the realm of science and technology throughout the 1980s [5]. The term “technology foresight” became widely recognized in the 1990s as a means of creating new policy tools to tackle issues within science, technology, and innovation frameworks [6].

Despite the wide array of available futures methods that could potentially benefit medicine and health care, very few of these attempts have received notable attention from the medical community. Some of these have been performed by consulting companies and agencies such as Deloitte and KPMG [7,8]. Others have been published by institutions such as the World Health Organization [9] and the European Commission [10]. Another prominent example is the so-called Topol Review that outlined recommendations to ensure the United Kingdom’s National Health Service is the world leader in using digital technologies to benefit patients [11]. It emphasized the implementation of technologies, such as genomics, digital medicine, artificial intelligence (AI), and robotics, in the future.

Given the recognition of such examples that might be limited in scope and impact, it becomes imperative to further explore the array of futures methodologies [1,12]. This exploration not only highlights the diverse techniques at our disposal but also underscores the untapped potential these methodologies hold in transforming health care planning and policy-making. Our scoping review reveals that the prevailing futures methods employed in the field of medicine and health care encompass trend analysis, Delphi, backcasting, policy analysis, technology assessment, horizon scanning, the Futures Wheel, and scenario analysis (Table 1). A concise overview of these methods is presented below.

**Table 1:** Comparison of the various foresight methods used in health care.

Foresight method	Description	Key characteristics	Common applications in health care
Trend analysis	Identifying patterns or trends in data over time	Data-driven, quantitative, predictive	Market trends, patient demographics
Delphi	Gathering expert opinions through iterative surveys to reach a consensus	Expert-driven, qualitative, consensus-building	Health care policy, clinical guidelines
Backcasting	Starting with a desired future and working backwards to the present	Goal-oriented, strategic, creative	Long-term health planning, sustainability initiatives
Policy analysis	Evaluating the effectiveness of policies and their potential impacts	Analytical, evaluative, policy-focused	Public health policies, regulatory changes
Technology assessment	Assessing the potential impacts of new technologies	Technology-focused, impact analysis	Adoption of medical technologies, risk assessment
Horizon scanning	Identifying emerging trends, threats, and opportunities	Forward-looking, broad-scope, proactive	Emerging health technologies, risk management
Futures Wheel	Analyzing the consequences of a particular event or statement	Brainstorming exercise	Direct and indirect impacts of technological, policy, or procedural changes
Scenario analysis	Exploring and evaluating possible future scenarios	Exploratory, strategic, scenario-building	Strategic planning, crisis management

Trend analysis refers to the prevailing directions or patterns of change and entails assumed developments in the future with a long-lasting effect and impact on a given field [13]. Trends often emerge from advancements in technology, shifts in patient needs and demographics, evolving health care policies, and new medical research findings. Such trends can be identified through data analysis, expert forecasts, and studying historical patterns in medicine and health care to help shape future strategies and provide insights into potential developments. Trend analysis is often applied to forecasting that is about generating predictions about the outcome of a specific question such as “How many mRNA-based vaccines will have been approved by the US Food and Drug Administration (FDA) by the end of 2030?”

The Delphi method was originally developed as a systematic interactive forecasting method, which relies on a panel of experts [14]. The use of Delphi is prevalent across health sciences research, and it is used to identify priorities, reach consensus on issues of importance, and establish clinical guidelines.

Backcasting is a method that starts with defining one or more preferred futures and then works backwards to identify strategies, policies, projects, or actions that will connect that specified future to the present [15]. Hence, it creates a roadmap toward the imagined future, such as how to effectively provide and organize future health care services.

Policy analysis examines problems calling for policy response and then proceeds to determine and assess possible options for policy action. Studying alternative futures and policy scenarios facilitates the better understanding of how present decisions affect the future [16]. It can indicate what measures improve the effectiveness and sustainability of health systems, or what priorities should be set for health-related research and development.

Technology assessment aims to examine the impacts of new technologies on society, including the capacity to be prepared for what may happen or be needed in the future. It anticipates the possible effects of technology-related evolution by collecting insights into the concerns of various stakeholders [17]. It offers a beneficial approach for health care manufacturers and decision makers to evaluate innovative health technologies prior to their launch. Considering that the successful launch of innovations in health care is substantially time and resource intensive, foresight-based strategic and future-oriented thinking is essential.

Horizon scanning provides strategic foresight through identifying emerging trends, technologies, and potential challenges in health care to anticipate future developments [18]. It involves gathering and analyzing data from a wide range of sources to spot early signs of significant change, also called “weak signals,” and evaluating the implications of these changes for health care systems and policies.

The Futures Wheel is a tool designed to help explore the potential consequences of a particular change or event. This structured brainstorming method, created by futurist Jerome C Glenn in 1971, visually maps out direct and indirect impacts, promoting a deeper understanding of the ripple effects that can arise from a single decision or trend.

Finally, scenario analysis involves creating and exploring multiple plausible future scenarios to understand the potential impacts of various trends, changes, and uncertainties in health care [19]. This could be useful in determining possible risks and opportunities as well as evaluating how resilient the current strategies are to various future circumstances.

Acknowledging the potential of using foresight in medicine and health care and seeing the fragmented attempts at exploiting the even higher potential of other futures methods, we aimed to explore this notion by undertaking the first scoping review to date about the application of futures methodologies and published foresight projects in health care.

## Methods

The first author (BM) and second author (TK) of this article performed a scoping review using the PRISMA-ScR (Preferred Reporting Items for Systematic Reviews and Meta-Analyses extension for Scoping Reviews) method [20]. The completed PRISMA-ScR checklist is provided in Multimedia Appendix 1. To avoid missing relevant papers, we used a search strategy covering 3 sources. The first included a broad search strategy focusing on futures methods and foresight on PubMed. The second source expanded that search to nonbiomedical journals by using Crossref. For the third source, we used the same search strategy in the 5 most influential (Scopus and Web of Science indexed) journals in the futures field, namely Technological Forecasting and Social Change, Futures, European Journal of Futures Research, Foresight, and Foresight and STI Governance. It is important to note that for now, futures studies and foresight are niche fields with a relatively small community of researchers and a limited number of influential journals, in contrast to larger disciplines. We considered the “most influential” journals to be those that are categorized as Scopus Q1 and are also indexed in Web of Science. The journals must focus specifically on futures studies or foresight. The sequence of mentioning the journals was driven by their latest available Scimago Journal Rank (SJR) index.

We focused on futures methods, studies, and foresight in the medical or health care–related context. As an initial filtering step, we decided to focus on select journal articles only. Articles not published in the English language were also excluded. Using the search query of (medicine OR medical OR health OR healthcare) AND (foresight OR futures studies OR futures methods OR futurology OR futurism), our initial search identified 448 publications in PubMed and 1301 publications in Crossref. Subsequently, to mitigate the risk of overlooking potentially pertinent future-oriented sources, we proceeded to employ an identical search query on the websites of the 5 aforementioned futures-related journals, resulting in 31 more records.

Throughout the subsequent screening process, Crossref hits were ranked in Google Scholar, and the first 100 items were selected for further analysis. The second author (TK) evaluated this list, conforming to the below outlined inclusion and exclusion criteria. Ultimately, 18 articles were selected from Crossref.

For PubMed, 403 records were excluded from the original list, and 45 records were checked for eligibility. Of these, 20 more were excluded. Therefore, 25 records were selected from PubMed.

Subsequently, the first author (BM) conducted a search within the previously mentioned 5 futures-related journals using the same keywords. Four relevant items were shortlisted from Technological Forecasting and Social Change, 4 from Foresight, 3 from the European Journal of Futures Research, 2 from Futures, and 1 from Foresight and STI Governance. To prevent duplication, articles already identified from Crossref were not selected again via journal search. Overall, 16 articles were selected for the final analysis from this search process.

We applied a range of inclusion and exclusion criteria to filter the literature. Articles were included if they met these criteria: (1) focus on foresight in medicine or health care, (2) original studies or reviews, and (3) use of recognized futures methodologies such as trend analysis, Delphi, backcasting, policy analysis, technology assessment, horizon scanning, or scenario analysis.

Articles were excluded if (1) the search terms were mentioned tangentially or in a context not related to futures in the fields of medicine and health care (eg, visual foresight in optometry); (2) the studies were not directly focused on futurist perspectives, even if they mentioned “foresight;” (3) the papers were not primary research articles or reviews, such as editorials, commentaries, book reviews, or news pieces; (4) the papers did not use or address recognized futures methodologies or the methodology was unclear or not well-defined; (5) the papers were from non-peer-reviewed journals as these might not meet the rigorous standards set by more reputable journals; and (6) the papers did not have at least an English abstract.

**Figure 1:**
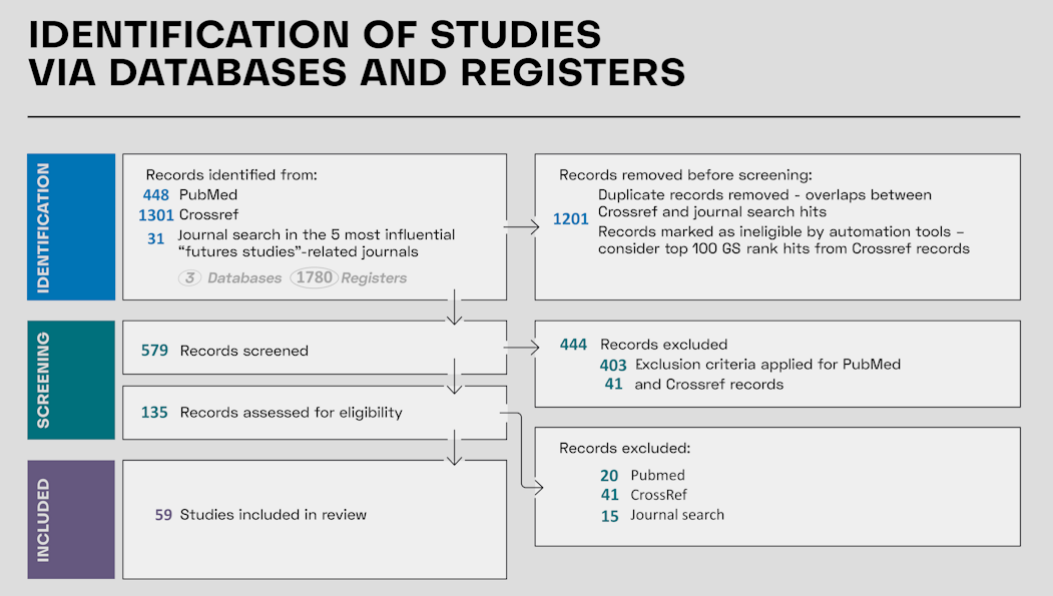
PRISMA-ScR (Preferred Reporting Items for Systematic Reviews and Meta-Analyses extension for Scoping Reviews) flowchart of the study selection process. The flowchart illustrates the study selection process for the scoping review. Initially, 1780 records were identified through database searches. After removing duplicates, title and abstract screening excluded 465 records, leaving 114 records for full-text assessment. Of these, 55 were excluded for not meeting the inclusion criteria, resulting in 59 studies for the final analysis. GS: Google Scholar.

We ended up including 59 articles in our review. Table 2 describes these articles briefly, while the extended description can be found in Multimedia Appendix 2. The PRISMA-ScR flow diagram illustrating this process is presented in Figure 1.

**Table 2:** Identified and analyzed articles.

Theme	Title	Author	Journal	Year	Methods/design
Health care policy and workforce dynamics	Management challenges for future digitalization of healthcare services	Gjellebæk et al [21]	Futures	2020	Scenarios, backcasting
Health care policy and workforce dynamics	Drivers, trends and scenarios for the future of health in Europe. Impressions from the FRESHER project	Wepner et al [22]	European Journal of Futures Research	2018	Scenarios, trend analysis
Health care policy and workforce dynamics	Rethinking health workforce planning: Capturing health system social and power interactions through actor analysis	Rees et al [23]	Futures	2018	Document analysis, semistructured interviews
Health care policy and workforce dynamics	Futures-oriented drugs policy research: Events, trends, and speculating on what might become	Rhodes et al [24]	International Journal of Drug Policy	2021	Speculative research/big event, mega trend
Health care policy and workforce dynamics	Looking at the fringes of MedTech innovation: a mapping review of horizon scanning and foresight methods	Gonzalez-Moral et al [25]	BMJ Open	2023	Horizon scanning, meta-analysis
Health care policy and workforce dynamics	An overview of future EU health systems. An insight into governance, primary care, data collection and citizens' participation	Quaglio et al [26]	Journal of Public Health	2018	Workshops, STI^a^ policy analysis
National strategies	Integrated foresight for the healthcare sector in Turkey	Kalan et al [27]	International Journal of Foresight and Innovation Policy	2007	Delphi, systems dynamics modeling
National strategies	The knowledge triangle in the healthcare sector — The case of three medical faculties in Norway	Brorstad Borlaug et al [28]	Foresight and STI Governance	2018	STI policy analysis
National strategies	Healthcare 2025: alternative views and underlying values	Rowley [29]	Foresight	2003	Scenarios
National strategies	Foresight in medicine. Lessons from three European Delphi studies	Wild et al [30]	European Journal of Public Health	2000	Delphi, meta-analysis
National strategies	The Dutch Public Health Foresight Study 2018: an example of a comprehensive foresight exercise	Verschuuren et al [31]	European Journal of Public Health	2019	Scenarios
National strategies	Forecasting Japanese health futures with the BFT	Watanabe et al [32]	Futures	1995	Forecasting
National strategies	The Futures Wheel model of the effects of the emerging infectious diseases pandemics on the elderly in Iran	Eidgahi et al [33]	Salmand	2024	Futures Wheel, expert panel, STEEP^b^ analysis
National strategies	Possible future scenarios of the general health social security system in Colombia for the year 2033	Aguilar et al [34]	European Journal of Futures Research	2023	Scenarios, in-depth interviews, system dynamics modeling
National strategies	Health-related R&D priorities until 2030: Russian experience	Saygitov [35]	Foresight	2017	Trend analysis, STI policy analysis
National strategies	Perceptions of future hospital management in Finland	Pihlainen et al [36]	Foresight	2019	Delphi, inductive content analysis
National strategies	Using foresight to explore the impacts of flooding in Houston on health, poverty, and equity out to 2050	Daniels et al [37]	Futures	2021	Delphi, cross-impact analysis, interviews
National strategies	Health scenarios and policy making: Lessons from the Netherlands	Schreuder [38]	Futures	1995	Scenarios
National strategies	Trend Impact Analysis (TIA) of community-based futures study for pediatric obesity in Iran	Taghizadeh et al [39]	BMC Pediatrics	2023	Trend impact analysis, Delphi
National strategies	The effect of coronavirus (COVID-19) pandemic on medical sciences education in Iran	Rezaei et al [40]	Journal of Education and Health Promotion	2021	Futures Wheel
National strategies	The future of AIDS in Africa: lessons from two scenario projects	Fourie [41]	African Journal of AIDS Research	2009	Scenarios
National strategies	The impact of future world events on Iranians’ social health: A qualitative futurology	Damari et al [42]	Iranian Journal of Public Health	2016	Cross-impact analysis, deep interviews
National strategies	Key factors in the future of oral and dental health in Iran using scenario writing approach	Mehrolhassani et al [43]	BMC Oral Health	2024	Scenarios, focus groups, cross-impact analysis
National strategies	Exploring the driving forces and scenario analysis for catastrophic and impoverishing health expenditures in Iran	Hedayati et al [44]	BMC Health Services Research	2024	Scenarios, systematic review
National strategies	Medical technology decisions in The Netherlands: How to solve the dilemma of technology foresight versus market research?	Postma et al [45]	Technological Forecasting and Social Change	2007	Delphi, scenarios, technological forecasting
Pandemic preparedness and response	How to anticipate and control possible future health crises through foresight approaches using the COVID-19 pandemic crisis?	Didier et al [46]	Archives of Community Medicine and Public Health	2023	Scenarios, VUCA^c^
Pandemic preparedness and response	Foresight in the time of COVID-19	Gariboldi et al [47]	The Lancet Regional Health – Western Pacific	2021	Think tank workshops, scenarios, backcasting
Pandemic preparedness and response	Preparedness for emerging infectious diseases: pathways from anticipation to action	Brookes et al [48]	Epidemiology and Infection	2015	Environmental scanning, horizon scanning, multi-criteria decision analysis, simulation modeling
Pandemic preparedness and response	Steering vaccinomics innovations with anticipatory governance and participatory foresight	Ozdemir et al [49]	OMICS: A Journal of Integrative Biology	2011	Anticipatory governance, participatory technology design
Pandemic preparedness and response	The importance of uncertainty in health scenarios: A scoping review on COVID-19 scenarios	Hosseini Golkar et al [50]	Medical Journal of the Islamic Republic of Iran	2023	Scenarios, systematic review
Pandemic preparedness and response	COVID-19 and the politics of hope: A comparative analysis of Greek and Ecuadorian letters from a desired post-pandemic future	Gross et al [51]	Futures	2023	Letters from the future, visioning, backcasting
Pandemic preparedness and response	The value of mass-produced COVID-19 scenarios: A quality evaluation of development processes and scenario content	Crawford et al [52]	Technological Forecasting and Social Change	2022	Scenarios, meta-analysis
Specialized medical domains	The impact of foresight studies on human healthcare in the post-genomic era	Damrongchai et al [53]	International Journal of Foresight and Innovation Policy	2010	Scenarios, Delphi
Specialized medical domains	Delphi in a future scenario study on mental health and mental health care	Bijl [54]	Futures	1992	Delphi
Specialized medical domains	Innovative application of strategic foresight to oncology research	Bishop et al [55]	Foresight	2020	Scenarios, framework foresight
Specialized medical domains	Future of bioprinted tissues and organs: A two-wave global survey	Mota et al [56]	Foresight and STI Governance	2022	Surveys
Specialized medical domains	Leveraging strategic foresight to advance worker safety, health, and well-being	Streit et al [57]	International Journal of Environmental Research and Public Health	2021	Three-horizon foresight (weak signals, seeds of change, trend analysis), scenarios
Specialized medical domains	Four futures for occupational safety and health	Felknor et al [58]	International Journal of Environmental Research and Public Health	2023	Scenario-based framework foresight
Specialized medical domains	How will the future of work shape the OSH professional of the future? A workshop summary	Felknor et al [59]	International Journal of Environmental Research and Public Health	2020	Workshops
Specialized medical domains	ASHP Foundation Pharmacy Forecast 2018: Strategic planning advice for pharmacy departments in hospitals and health systems	Vermeulen et al [60]	American Journal of Health-System Pharmacy	2018	Surveys, collective wisdom of “wise crowds”
Specialized medical domains	Personalized medicine beyond genomics: alternative futures in big data-proteomics, environtome and the social proteome	Özdemir et al [61]	Journal of Neural Transmission	2017	Technology foresight analysis
Specialized medical domains	Synergy between competitive intelligence (CI), knowledge management (KM) and technological foresight (TF) as a strategic model of prospecting--the use of biotechnology in the development of drugs against breast cancer	Canongia [62]	Biotechnology Advances	2007	Competitive intelligence, technology foresight
Specialized medical domains	The future of ICT for health and ageing: Unveiling ethical and social issues through horizon scanning foresight	Flick et al [63]	Technological Forecasting and Social Change	2020	Horizon scanning

^a^STI: science, technology, and innovation.

^b^STEEP: social, technological, economic, environmental, and political.

^c^VUCA: volatility, uncertainty, complexity, and ambiguity.

The following data were extracted from each included study: title, authors, journal, year of publication, summary, methods/study design, whether the study is available in open access, and access URL. These data were entered into a Microsoft Excel spreadsheet for analysis. The corresponding author (BM) performed the initial analysis, and the second author (TK) cross-checked it.

## Results

### Themes

We identified the following 5 themes and assigned each selected study to one of those themes (Table 2; Multimedia Appendix 2):

1. National strategies (n=19): These studies focus on the unique health-related prospects, challenges, and trends within specific countries. They offer insights tailored to each nation’s health care system, demographics, and sociocultural milieu.
2. Strategic health care foresight (n=15): These studies employ futures methodologies to provide high-level analyses of future medical scenarios, trends, and challenges. They are broad in their approach, often looking at overarching factors that may influence the medical field as a whole.
3. Health care policy and workforce dynamics (n=6): This theme encompasses studies that focus on the impact of governmental policies on health care as well as the dynamics of managing health care professionals. They explore strategies and potential reforms that could shape the future of medical governance and human resource management.
4. Pandemic preparedness and response (n=7): These studies address issues related to infectious diseases, with a particular emphasis on vaccination strategies and the challenges posed by the COVID-19 pandemic. They discuss potential future scenarios, challenges, and strategies related to managing and preventing infectious disease outbreaks.
5. Specialized medical domains (n=12): This theme centers on specific areas or disciplines within medicine, such as personalized medicine, oncology, or biotechnology. Papers under this category delve deeply into their respective niche, highlighting future challenges and opportunities.

### Theme 1: National Strategies

In this section, we present key findings from a selection of studies that illuminate the integration of futures methodologies in national health care strategies. These studies collectively highlight the growing recognition of foresight as a useful tool in shaping the future of health care policy and practice.

Iran clearly stands out in terms of foresight and futures studies as 6 studies focused on Iranian perspectives. Rezaei et al [40] used the Futures Wheel method to identify the effects of COVID-19 on medical sciences education. Nejatzadehgan Eidgahi et al [33] used the same technique to understand the different effects of emerging infectious disease pandemics on elderly people based on the experiences from the COVID-19 pandemic. Damari et al [42] performed a qualitative study of futurology with cross-impact analysis to find potential domains that may have an impact on Iranians’ social health. Mehrolhassani et al [43] conducted a study in 3 stages, including a scenario writing approach, qualitative methods, and exploratory future research, to analyze the future of oral and dental health. Moreover, Hedayati et al [44] conducted a literature review and obtained experts’ opinions using the Scenario Wizard software to provide insights into potential future scenarios of health expenditures in the country. Taghizadeh et al [39] conducted a community-based futures study using trend impact analysis on pediatric obesity, accentuating the effectiveness of foresight in policy evaluation, localization, and communication for obesity prevention.

Kalan et al [27] introduced an integrated foresight process within Turkey’s health care sector, exemplifying the role of foresight in creating reflexive knowledge, fostering futures literacy, and developing strategic plans. The study provided evidence to promote futures literacy that helped define the causal relationships in the sector to elaborate and accomplish strategic action plans. At the same time, Postma et al [45] proposed a similar approach involving a method merging expert opinion forecasts with market-oriented scenarios in a Dutch case study on cancer imaging techniques, addressing the dilemma of short-term market perspectives versus long-term technology foresight.

Interdisciplinary approaches were highlighted in 2 studies. Verschuuren et al [31] presented the Dutch Public Health Foresight Study 2018, emphasizing a multidisciplinary participatory approach to explore the future of public health and health care in the Netherlands. It combined quantitative scenarios with qualitative thematic studies and participatory stakeholder consultations. The results indicated that well-established decision-making for the future requires an interdisciplinary approach, which is essential in completing complex foresight projects. Similarly, Daniels et al [37] employed foresight to examine the impact of severe flooding on health, poverty, and equity in Houston. They presented plausible scenarios to engage stakeholders and policymakers in shaping an inclusive future for the city’s residents. The study concluded that foresight is a critical competence that equips practitioners with the ability to anticipate the implications of decisions.

Two papers focused on specific communities. Saygitov [35] provided insights into Russian efforts to establish research priorities in the health sector and contribute to health-related research and development and innovation policies through the use of trend analysis and expert workshops. UNAIDS and the South African financial services group Metropolitan published a set of scenarios regarding the future impact of HIV/AIDS in Africa and South Africa, respectively [41]. The paper highlighted the many lessons to be gleaned for HIV-related health planning and policy-making in general.

Wild et al [30] examined 3 technology foresight exercises (Delphi studies) focusing on health care innovations, drawing trends from German and British Delphi approaches and a more focused Austrian Delphi. The paper concluded that health policy must not only respond to these innovations but also proactively guide them, emphasizing the need for cooperation and coordination among all stakeholders. Hence, it is an important purpose and responsibility of futures studies, including in medical foresight, to enhance democratic participation, as participating actors have a key role in shaping the future. Pihlainen et al [36] explored Finnish experts’ perceptions of hospital management and leadership for the year 2030, using a 3-round Argument Delphi process. The results of the foresight exercise contributed to the development of management and leadership training and future-oriented management practices in hospitals.

Value-driven scenarios dominated the rest of the papers in this theme. Brorstad Borlaug et al [28] explored the significance of education in the Norwegian health care sector, highlighting the interplay between education, research, and innovation, and its implications for national health care policy. Rowley [29] employed scenarios to initiate public dialogue on health care values in the United States, highlighting the role of values in shaping diverse health care futures and providing a framework for evaluating value-driven components. It is also revealed that futures methods nowadays are able to evaluate values in an objective and future-oriented manner. Aguilar et al [34] formulated future scenarios for the Colombian General Health and Social Security System in 2033, promoting anticipatory governance and participatory policy development.

Another paper shared lessons of the scenario projects the Dutch government’s Steering Committee on Futures Health Scenarios organized and carried out to facilitate research and debate on alternative futures in public health [38].

Another example demonstrated the power of forecasting. Japan’s Institute of Health Systems Development created Bioforecasting Technology that involves integration of data regarding the population, the natural and social environment, and the health, medical, or social services with projections on the future population’s demand for different kinds of health services. They claim that its forecasts have proved to be highly accurate [32].

### Theme 2: Strategic Health Care Foresight

Studies that explore the strategic application of futures methodologies in the health care industry are presented in this section, with an emphasis on a range of topics such as ethics, innovation, sustainability, and participatory approaches.

Hemmat et al [64] reviewed and compared research on the future of health IT. The study concluded that while forecasting is suitable for smaller objectives, foresight approaches are better for achieving larger-scale goals. It helps establish reasonable long-term expectations for improvements in health IT. Furthermore, the use of mixed methods in foresight projects can better illuminate the necessary actions to accomplish preferred futures, whereas forecasting was found to be more theoretical than practical.

Mittelstadt et al [65] conducted a systematic meta-analysis of the academic literature to understand the ethical implications of “big data,” especially in the biomedical field. It identified 5 primary areas of ethical concern and highlighted 6 additional emerging areas needing attention. This framework aims to guide future ethical assessments and governance of big data practices, especially in the very sensitive context of biomedical research.

With respect to upcoming innovations in health, Butter et al [66] evaluated the degree of alignment between the EU Framework Program (FP7) and the foresight community. Using the Dynamo approach, the study analyzed 140 flagship foresight activities and offered insights for the foresight community to prioritize unaddressed health innovation themes.

Masum et al [67] used compelling cases to highlight the usefulness of 5 futures methods (forecasting, scenario planning, Delphi, technology road mapping, and mass collaboration) in medicine and global health decision-making. It provided evidence that applying futures methods to health can help prepare for the future, including policy development and health initiatives.

Kulkova et al [68] focused on identifying key and atypical factors influencing the development of medicine, particularly in light of the challenges posed by COVID-19. The study, which employed the Delphi method, identified well-being technologies, data-informed personalization, and climate change as the 3 main drivers for the next 1 to 50 years. The results point out the processes that will influence the industry’s transformation and suggest that stakeholders take appropriate measures to respond organically to events.

From a multi-stakeholder standpoint, Pereno et al [69] addressed the future of sustainable health care with the goal of defining long-term scenarios and key strategies for transitioning to sustainable health systems. It involved a collaborative foresight process through workshops with stakeholders from the Nordic countries, including health industries, providers, managing authorities, universities, research centers, nongovernmental organizations, and health care networks. The study identified 3 future horizons and drivers for reshaping stakeholder roles, underlining sociotechnical transition toward collaborative, distributed dynamic networks.

In order to envision health care futures in 2040 and beyond, Pau et al [70] investigated a methodology for participatory futures studies involving nonexperts, particularly in contexts with extreme unknowns. Rather than focusing on opinion leaders or scenario writers, the article highlights the role of futurists and foresight practitioners as facilitators.

Lamé et al [71] examined the use of scenario planning in the health care sector, focusing on a project at a large hospital’s cancer division. The authors proposed directions for future scenario planning in hospital settings and for research on matching scenario approaches with specific application contexts.

Using science, technology, and knowledge indicators, Antunes et al [72] presented a methodology for technological foresight and scanning with a focus on health care. The approach incorporates indicators involving patents (technology), research and development (science), and competencies (human resources) through international databases and a data-and text-mining tool to create knowledge maps for decision-making support.

Karlsen et al [73] reported on 20 experts envisioning the future of addictions and lifestyles in Europe by 2030 and beyond. The project used a combined approach, blending in-situ idea generation with established futures tools, which culminated in a 2-day electronic scenario workshop. Using backcasting, the experts proposed an optimistic outlook for Europe’s future addictions and lifestyles, focusing on collective values, long-term planning, and restitutive solutions to reframe current drug disorder policies.

Five articles focused on a more high-level approach about using futures methods in health care. Bezold [74] described a special issue of Futures, reflected on the significant growth of health futures, and argued that the future of health futures will be shaped by the ability of futures techniques and those who use them to provide value to organizations and individuals. Pau et al [70] described the importance of long-term thinking in health care through presenting an 18-month design research project that developed a methodology for participatory futures studies with nonexperts. Haghdoost et al [75] contemplated about the emergence of futures methods in health care and concluded that there is a lack of a unique and dominant discourse in health sciences around those methods. Hosseini Golkar et al [76] analyzed the usefulness and importance of scenario planning and health scenario writing for short- and medium-term futures. Finally, Doos et al [77] performed a systematic search to identify the methods used in forecasting studies to predict future health technologies within 2 decades.

### Theme 3: Health Care Policy and Workforce Dynamics

This theme’s studies explored the dynamics of health care policy and employed futures methodologies to prepare the workforce for the future.

Rhodes et al [24] discussed the need for a more speculative approach in drug policy research, moving beyond predictable, probability-based analyses to embrace possibilities. The authors outlined the use of “Big Event” and “Mega Trend” as tools for speculative intervention, helping to trace and project complex factors influencing drug futures. The aim of this approach was to influence the present about what futures could be.

In order to identify horizon scanning and other approaches used for medical technology foresight in health care decision-making, Garcia Gonzalez-Moral et al [25] conducted a review. It revealed a diverse range of methods, highlighting the need for greater transparency and consistency in methodology reporting. The results shed light on the fact that available methods used in combination can overcome the limitations posed by single methods and no best practice exists in this regard.

Quaglio et al [26] summarized the outcomes from a European Parliament workshop titled “Health systems for the future,” where experts and decision-makers discussed enhancing the effectiveness and sustainability of EU health systems. The paper concluded that in order to equip EU health systems to handle upcoming challenges, leadership in policy-making will be essential.

Gjellebæk et al [21] explored middle management strategies for promoting workplace learning in the context of introducing eHealth in health care. The study established the need for a shift toward learning-oriented leadership and adaptive management, focusing on employee involvement and continuous skill development. Scenario planning and backcasting were found to be effective tools for navigating the complexities of changing work practices.

Rees et al [23] demonstrated the importance of actor analysis for identifying strategic positions on health workforce planning, acknowledging the need for accommodating changes in health care roles and environments. The study employed a mixed methods design, combining document analysis, semistructured interviews from 2 health subsectors, and numerical data transformation for actor analysis software.

Wepner et al [22] considered stakeholders from a range of sectors as they investigated alternative policy approaches for noncommunicable diseases in OECD (Organization for Economic Co-operation and Development) countries. The study analyzed trends to develop 4 scenarios of possible futures. The article concluded that out of the box thinking is needed to consider the complexity of future health systems that need to include aspects like equity, literacy, mobility, or urban planning. It makes the case for a systematic and holistic approach to address all drivers and determinants leading to a healthy life and well-being.

### Theme 4: Pandemic Preparedness and Response

This theme highlights studies that focused on the application of futures methodologies in pandemic preparedness and response.

The application of futures methodologies in the Western Pacific region to enhance public health emergency response, specifically for the COVID-19 pandemic, was detailed by Gariboldi et al [47]. They used agile think tank sprints, a 6-step foresight process, and backcasting to develop pandemic scenarios over an 18-month horizon, resulting in tailored recommendations for World Health Organization response and country support, and demonstrating foresight’s value in public health emergencies. Through the creation of diverse future scenarios that considered not only predictable trends but also areas of uncertainty and divergence, potential shocks to the system, and complex interactions, a more future-proof strategy could be devised.

In their review, Brookes et al [48] suggested integrating tools for identifying, prioritizing, and investigating emerging and re-emerging infectious diseases into a comprehensive framework to improve tactical and strategic planning for emerging risk preparedness. Techniques included environmental scanning, foresight programs, horizon scanning, surveillance, transparent prioritization methods, risk assessment, and simulation modeling. The results suggested that the coordinated application of multiple foresight tools provides a greater overall benefit than individual tools applied in an ad hoc manner.

Since anticipatory governance is becoming increasingly crucial for responsible innovation and scientific practice, particularly in the uncertain and high-stakes field of vaccinomics, Ozdemir et al [49] argued in favor of its application with foresight. This article corroborated one of the core tenets of futures studies, namely that uncertainty is not an accident of the scientific method but is its very substance. Anticipatory governance with participatory foresight offers a mechanism to respond to such inherent sociotechnical uncertainties in the field of vaccinomics by reflective generation of futures literacy.

The evolution of prospective studies toward an integrated, diachronic approach to envisioning the future, especially in uncertain situations like the COVID-19 crisis, was analyzed by Didier et al [46]. It highlighted foresight as a discipline that explores possible futures and informs present choices to shape desirable futures and strategize against unwanted scenarios, particularly in health-related contexts like pandemics and epidemics. The core message of the article lies in the finding that foresight creates a space for a wide range of dialogues and facilitates thinking ahead about the actions that must be taken now to achieve the world we prefer in the future.

Crawford et al [52] evaluated 213 COVID-19 scenarios developed during the pandemic’s first wave, addressing concerns about the value of these mass-produced scenarios. The study concluded that scenario interventions, particularly in crises, should be more proactive and inclusive of stakeholders, decision-makers, and affected communities to ensure effectiveness and relevance.

Gross et al [51] conducted a unique experiment in which they invited people living in countries in Europe, North and South America, Asia, and Africa to imagine what the future could look like, beyond the COVID-19 pandemic, by writing a letter from the future. In their study, they analyzed those letters to reveal a particular relationship between the present and the future: a relationship of hope.

Hosseini Golkar et al [50] examined the phenomenon of uncertainties regarding the COVID-19 pandemic in relation to scenario planning. They made recommendations about improving the accuracy of scenario planning.

### Theme 5: Specialized Medical Domains

This theme features studies demonstrating the use of futures methodologies in specialized medical domains, including occupational safety and health (OSH), pharmacy practice, psychiatry, or breast cancer drug development.

Streit et al [57] examined the use of strategic foresight as a tool to address the evolving factors in OSH related to the workforce. The study reviewed work-related future scenarios, presenting a tailored foresight framework for OSH. The article paved the way for the integration of strategic foresight into OSH research and practice to enhance worker safety, health, and well-being. Generating the implications of plausible work-related futures can help OSH prepare for, plan, and influence the future by combating human tendencies to over- and underpredict change, which are critical errors in decision-making.

Felknor et al [58] detailed the US National Institute for Occupational Safety and Health inaugural strategic foresight project aimed at enhancing the OSH response to rapid workplace changes. Using futures studies and strategic management, the project developed 4 alternative future scenarios for OSH through multidisciplinary expert collaboration. This study provided evidence that widely used corporate foresight approaches can be successfully adopted in other fields.

In another article, Felknor et al [59] summarized a workshop focusing on the future of work and its implications for OSH education and training. The workshop featured national experts and thought leaders in OSH, strategic foresight, systems thinking, and industry, addressing challenges and opportunities in both academic and industrial contexts. The main point is that a transdisciplinary perspective should be applied to incorporate multiple disciplines, professions, and technologies into OSH academic training.

The sixth edition of the Pharmacy Forecast report, which emphasizes its role in advancing pharmacy practice leadership, was presented by Vermeulen et al [60]. This edition benefitted from contributions by pharmacists, advisors, panelists, and chapter authors. It also offered insights into emerging trends in pharmacy and health systems, aiming to stimulate strategic planning rather than making precise predictions.

Özdemir et al [61] discussed the integration of big data technologies like proteomics in psychiatry, emphasizing its role as a next-generation biomarker and diagnostic tool. The article proposed a 3-tiered nested governance structure for big data science, involving scientists, ethicists, and scholars in ethics of ethics, to ensure equitable distribution of political power in innovation processes.

With a focus on biotechnology for breast cancer drugs, Canongia’s paper [62] presented a strategic model that combines technology foresight, competitive intelligence, and knowledge management to support decision-making in sustainable development and innovation. The findings showed that technological foresight has a common base in the process of gathering strategic information, the implementation of analytical and reflective capacities, and the creation of futures literacy.

Damrongchai et al [53] evaluated the impact of foresight on policy, demonstrating its use through a case study on health care in the postgenomic era. The study highlighted foresight as a crucial tool for understanding the complexity of potential impacts, reconciling different perspectives, and forming a solid foundation for policies, with assessment of the project’s impact against various future outcomes.

Horizon scanning was used by Flick et al [63] to explore the future of information and communication technologies (ICT) for health and aging, with a particular focus on the ethical and social issues in this sector. In order to discuss the potential and likely futures of ICT for health and aging, the study analyzed interviews, literature reviews, policy documents, and online public discussions to identify key and weak signals in areas such as future technologies, companies, older people, and environments.

With a focus on personalized medicine, Stelzer et al [78] introduced a novel method that combines bibliometrics and the scenario technique for technology foresight. The method involves an 8-step scenario approach, incorporating emerging technologies and their effects on each scenario, supported by co-citation analysis and a bibliographic coupling network. The findings show that by addressing each other’s shortcomings, these 2 approaches together produce a helpful, all-around tool for conducting technology foresight.

Bishop et al [55] applied Framework Foresight, a method from the University of Houston, to explore the future of cancer and cancer research for strategic planning in a cancer research center. It identified 4 scenarios that highlighted strategic issues and offered guidance for not only cancer centers but also medical practice in general. This paper marks the first application of Framework Foresight to a medical topic. As the University of Houston is currently the world leading higher education institution in the field of futures studies, this case study demonstrates fruitful cooperation among academic futurists, foresight practitioners, and stakeholders from the medical sector.

Experts in tissue engineering from around the world were surveyed in 2 stages by Mota et al [56] on the potential applications of 3D and 4D bioprinting in preclinical research and clinical settings. The study also proposed a practical tool for expert identification and interviewing, which is useful for foresight studies. The results revealed that the influence of technology on the health sector tends to further increase over time. Henceforth, preparing for the future is a necessity for not only those involved in research, clinical practice, or technology management but also those responsible for health care delivery and developing and implementing public health policies.

As the final example of applying futures methods to health care in this theme, Bijl [54] highlighted the benefits of the Delphi method for analyzing issues of futures research on mental health and its potential impact on policy-oriented futures research.

## Discussion

### Principal Findings

This investigation not only demonstrates the various futures methods available to the medical community but also emphasizes the unexplored possibilities these approaches possess in transforming health care planning and policy formulation.

The studies in the first theme collectively demonstrate how futures methodologies are becoming more widely acknowledged as crucial instruments in shaping national health care strategies and policies, with implications for future health care practices and innovations.

Studies under theme 2 highlight the strategic applications of futures methodologies in health care, ranging from ethics and innovation to sustainability and participatory approaches, enriching the field with diverse perspectives and insights.

Theme 3 addresses the complexities of preparing the health care workforce for future challenges. Studies under this theme offered insights into health system transformation, the training of medical professionals, actor analysis, and policy alternatives for future health care implications.

Studies under theme 4 underscored the significance of using futures methodologies in pandemic preparedness and response, offering insights into their application in public health emergencies, which is a timely subject owing to COVID-19’s impact on health care.

The fifth theme demonstrated the wide-ranging applications of futures methodologies in specialized medical domains, from fostering innovation and strategic planning to helping health care professionals make well-informed decisions.

It is possible to conclude from the abovementioned narratives that “futures studies” has been used either sparingly in the health care industry, concentrating only on specialized areas and pandemic preparedness, or infrequently, addressing topics related to national strategies or the health care workforce. Although we managed to identify signs of various futures methods being used to anticipate health care futures, scenarios, and potential challenges, their theoretical and methodological underpinnings are not yet comprehensively developed.

In summary, our scoping review found that the use of futures methods has been successfully demonstrated in a wide range of fields from national strategies and policy-making to preparing for global threats and technological breakthroughs. Overall, 8 futures methods, including trend analysis, Delphi, backcasting, policy analysis, technology assessment, horizon scanning, the Futures Wheel, and scenario analysis, have already been used in medicine and health care, whereas over 50 futures methods exist. It may underscore the notion that the field is unexploited. These findings demonstrate that there are unmet potentials in the field, and therefore, we advocate for a broader interdisciplinary approach to future-oriented studies in medicine and health care.

We argue that these findings create an imperative need for the establishment of “medical futures studies” as a distinctive subdiscipline within the expansive domains of health care, medicine, and life sciences. This pioneering branch is envisioned to be committed to the systematic application of futures methodologies, with the primary objective of proactively anticipating and mitigating potential challenges, deliberating on policy considerations, and discerning emerging technological trends within the health care sector. This inclusive term could encompass foresight, futures methods, and practices, effectively bridging strategic planning with academic exploration.

This is a result of our realization that “futures studies” in this field is currently underutilized, which we consider to be a lost opportunity. We propose that the integration of futures studies has the potential to greatly enhance and advance medical science by filling a crucial gap in accomplishing well-founded anticipation, innovation, futures literacy, scenario formulation, visioning, and strategic planning.

**Figure 2:**
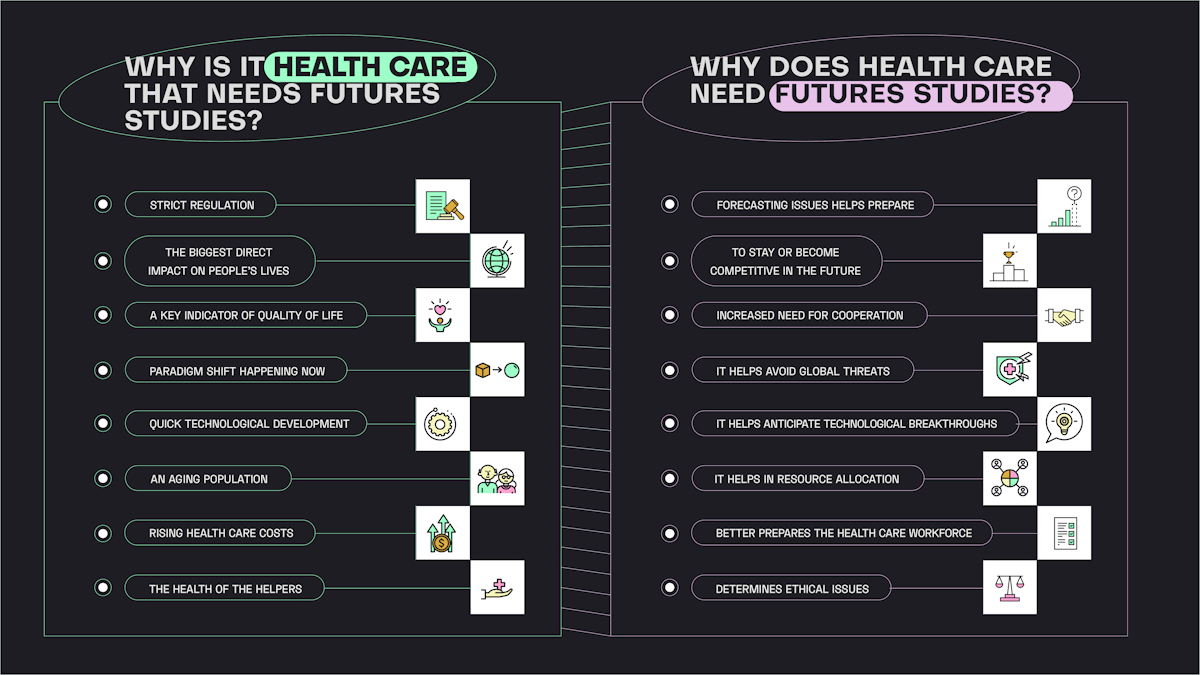
A summary of the reasons why health care is the prime candidate for the incorporation of futures studies and why the use of futures methods would be paramount for the evolution of health care.

We further elucidate why, among a multitude of fields, health care stands as the prime candidate for the incorporation of futures methods, and conversely, why futures methods are paramount for the evolution of health care (Figure 2).

First, we elaborate on why *health care is the prime candidate for the incorporation of futures studies*. Since health care regulations are uniquely stricter than those in other fields, it would be beneficial for regulators to anticipate challenges and benefits of changes in science, technology, and culture. In this manner, innovations could reach the market as a result of prompt and suitable regulations. The US FDA regulating AI in the health care sector is an exemplary story to underscore this [79]. It should be highlighted that one of the most important purposes of futures studies is to develop reflexive futures literacy and create knowledge about the future in order to underpin policy alternatives and decisions in such a way as to shape the future and promote the realization of preferred futures.

Health care has the biggest direct impact on people’s lives. From physical, mental, and emotional well-being and longevity, access to quality health care directly affects a person’s lifespan [80]. Moreover, good health enables people to work, contribute to the economy, and pursue their aspirations. Besides these observations, effective health care systems monitor and control the spread of infectious diseases. It is naturally supported by futures studies as its general mission is to contribute to the creation of a better, more livable world for human beings within the framework of a forward-looking approach.

Health care is also a key indicator of quality of life. According to Britannica, it is “the degree an individual is healthy, comfortable, and able to take part in or enjoy life events” [81]. Without good health, individuals may find it challenging to work or pursue education. Good health, supported by quality health care, allows individuals to engage in social activities, foster relationships, and participate in community events.

Health care’s biggest paradigm shift is happening in this decade [82]. Paradigm shifts have taken place in every industry. In the media, there has been a shift from print to digital platforms. In retail, there has been a shift from stores to e-commerce. In transportation, there has been a shift from gasoline to electric vehicles. Health care’s paradigm shift called digital health, during which patients become the point-of-care and the newest member of the medical team, has been taking place during recent years.

Consequently, an incredibly quick technological development is happening in health care that also comes with a cultural revolution. Medicine is highly dependent on technology, and the pace of technological advancement in health care is staggering. Futures methods can help anticipate the impact of emerging technologies, such as AI, gene editing, and telemedicine, on health care delivery, patient outcomes, and the overall health care landscape.

As the global population ages and health care needs become more complex, there is a growing need for innovative approaches to address the demographic challenges associated with elderly people. Research with an eye toward the future can help in envisioning the health care needs of aging populations and developing strategies to meet those needs effectively.

Health care costs are a significant concern worldwide, leading to one of the biggest challenges in the 21st century. Future-oriented research can explore ways to make health care more efficient, cost-effective, and accessible.

The health of helpers is also an important aspect. It is crucial to underscore the documented negative impact on a patient’s recovery when health care professionals are overworked or experiencing burnout. By conducting future-oriented research, we can explore innovative approaches to alleviate the burdens on health care professionals, promote their well-being, and ensure they can provide optimal care to patients. Moreover, addressing health care worker burnout benefits not only patients but also health care practitioners themselves. It can make the health care profession more enjoyable, fulfilling, and effective. This can lead to increased job satisfaction, reduced turnover rates, and a more motivated and engaged health care workforce.

We also describe why *the use of futures methods would be paramount for the evolution of health care*.

Anticipating issues and challenges would be of value for health care to better consider the advantages and disadvantages of technologies. Forecasting has been used for that purpose, among other things, in economics but has not been widely used in health care. As much as traditions are comfortable and give a sense of security, the emergence of evidence-based medicine and the need for scientific rigor marked a significant paradigm shift in the realm of medicine. Information has emerged as an invaluable commodity, the cornerstone of research, and the basis for every treatment decision. However, as important as it is, it is anchored in the present cross-sections. The dynamic nature of health care, which involves societal changes, unprecedented technological advances, and global events, yearns for a forward-looking perspective that futures studies can offer.

Foresight exercises can reveal that the challenges facing health policy involve not only responding to technological innovations by offering a universal promotional environment, but also steering and guiding them in a desirable future direction at an early stage. It could help detect high-level trends for decision makers, such as patients, becoming the point-of-care through a digital health paradigm shift, the art of medicine arriving with the era of AI, or digital health being a cultural transformation [83]. Understanding high-level trends might put decision-makers in a better position to foresee challenges, risks, potentials, and issues that might not yet exist.

Futures studies could help avoid global threats or help prepare in time for global threats. The emergence of new diseases, epidemics, or pandemics poses a significant risk to global public health. Medical futures studies can help anticipate and prepare for these threats, allowing governments and organizations to develop prevention and response strategies that minimize the impact on public health. A Canadian AI technology providing alerts of a possible outbreak in the early days of COVID-19 before national public health authorities did is a prime example of that [84].

In addition, futures studies can help anticipate and prepare for breakthroughs in medical technologies, such as gene editing, personalized medicine, and AI-driven diagnostics. By doing so, the medical community can ensure the readiness of infrastructure, policies, and regulatory environments for seamless adoption of these innovations. Medical associations have been trying to benefit from that [85].

Health care professionals can be better prepared for the quickly changing medical landscape, stay on the cutting edge of knowledge and practice throughout their careers, and identify new critical skills like prompt engineering by incorporating futures studies into medical education [86]. Current medical curricula run the risk of becoming outdated and sticking to less effective and cumbersome practices. The ways students now consume and understand subjects have evolved, with short-format videos, engaging AI or augmented reality projections, virtual reality, and AI-driven patient simulations taking precedence over traditional textbooks and quizzes. The new curriculum should also familiarize students with the basics of digital health and address the growing needs of patients in the face of a decreasing health care workforce. The imminent ubiquity of AI in medical practice means that medical professionals unequipped with these tools risk lagging in their field. Futures studies can provide solutions to these challenges and clarify whether they relate to the evolving dynamic between patients and doctors or the need to equip medical professionals with the digital skills of the future.

Foresight exercises on health technologies can also facilitate envisioning technological developments in order to stay or become competitive in the future. This can be relevant to not only companies trying to contribute to the progress of medicine but also individuals working in and for health care.

Future health challenges are becoming complex and require intersectoral solutions. With an emphasis on broad stakeholder engagement and the integration of varied forms of evidence to undertake collective visioning, futures approaches can strengthen long-term capacities for ongoing participatory evidence-based decision-making.

Moreover, technological innovations emerge from the imagination and skills of individuals acting in an appropriate setting. Foresight exercises identify common grounds of experts from different fields with respect to their understanding of future developments in their profession on a broad base, including policy options. In the future, there will be an increased need for cooperation and coordination among all actors in health care.

Futures studies can also help health care providers and policy-makers to optimize resource allocation by forecasting the demand for health care services and identifying potential bottlenecks or shortages in care [87].

Lastly, applying foresight could aid in determining ethical issues. As advancements in medical technology raise ethical concerns, medical futures studies can help anticipate and address these issues proactively. By engaging in ethical debates early on, the medical community can ensure responsible development and use of new technologies.

An obvious follow-up to this scoping review could be performing systematic reviews of the abovementioned futures methods individually to determine and demonstrate their use cases, benefits, and limitations specifically in medicine and health care.

### Limitations

This study has some limitations. We may have missed studies dedicated to foresight and futures methods as such scientific studies are spread across different types of journals ranging from biomedicine to business management. The nomenclature also varies as some studies use foresight, while others might refer to the same methods using other expressions such as futurology, futuristics, or futurism.

As many of the identified studies used different futures methods, it is difficult to compare them to each other. As we only focused on health care and the potential of futures studies to be used in that field, we might have missed other niche areas where futures research could also fill important gaps.

### Conclusion

Based on the findings of this study, we conclude that there is an imperative need for the introduction of medical futures studies. In addition to helping policy makers and medical professionals better anticipate emerging technological trends, breakthroughs, challenges, and ethical considerations as medicine advances, a systematic and guideline-based application of futures methods could close significant gaps in health care. We argue that launching a dedicated scientific journal in medical futures studies to foster a new research community, appointing dedicated futurists in decision-making and national strategy, and teaching basic futures methods in the medical curriculum could provide further contribution.

In this study, we conclude that embracing medical futures studies in health care is essential for guiding advancements, informing policy, and shaping education, ultimately leading to a more prepared, innovative, and ethical medical landscape.

## Appendix

Multimedia Appendix 1 PRISMA-ScR (Preferred Reporting Items for Systematic Reviews and Meta-Analyses extension for Scoping Reviews) checklist.

Multimedia Appendix 2 Extended database of the identified and analyzed articles.

## Abbreviations

AI: artificial intelligence
FDA: Food and Drug Administration
ICT: information and communication technologies
OSH: occupational safety and health
PRISMA-ScR: Preferred Reporting Items for Systematic Reviews and Meta-Analyses extension for Scoping Reviews

## Data Availability

All data extracted from the analyzed studies are available in the Multimedia Appendix section.
